# Editorial: Ancient diseases and medical care: paleopathological insights, volume II

**DOI:** 10.3389/fmed.2025.1672073

**Published:** 2025-08-18

**Authors:** Andreas G. Nerlich, Raffaella Bianucci, Francesco Maria Galassi, Sahar N. Saleem

**Affiliations:** 1 Department of Forensic Histology, Paleopathology and Mummy Research, Institute of Legal Medicine, Ludwig-Maximilians University, München, Germany; 2 Laboratoire Anthropologie Archéologie Biologie (LAAB), UVSQ/Université Paris-Saclay, UFR Simone Veil - santé, Montigny-le-Bretonneux, France; 3 Department of Anthropology, Faculty of Biology and Environmental Protection, University of Lodz, Łódz, Poland; 4 Department of Radiology, Kasr Al Ainy Faculty of Medicine, Cairo University, Cairo, Egypt

**Keywords:** paleopathology, paleoradiology, ancient medicine, cancer, malaria, brucellosis, mummy

The Research Topic “*Ancient diseases and medical care: paleopathological insights, volume II*” in Frontiers in Medicine emerges as a vibrant and multidisciplinary platform where history, archaeology, biomedical science, and paleopathology converge to elucidate the dynamic landscape of disease and healing across civilizations. This is the continuation of volume I which had provided a very successful compilation of highly recognized papers in the field (1). The five contributions selected for this volume are emblematic of the field's ongoing commitment to expand the evidentiary and methodological base of paleopathological inquiry. It is therefore encouraging to see that historical, paleopathological and molecular approaches converge into a common concept of research.Together, they offer a composite view of how disease shaped, and was shaped by, human behavior, environment, medical practices, and cultural translations over millennia.

A recurring theme throughout the issue is the notion of medical frontiers: boundaries of knowledge, diagnostic ability, and therapeutic innovation—whether breached, glimpsed, or defined—in ancient and early modern societies. From the analysis of long-forgotten fevers in Corsica to cancer management in Pharaonic Egypt, each article underscores the growing capacity of modern science to decode past lives and medical experiences through cutting-edge imaging, molecular biology, and historical linguistics. At the same time, these studies reflect how ancient societies—in diverse ways—actively conceptualized and responded to disease, sometimes in ways not unlike our own.

The study by Boualam et al. on malaria in medieval Corsica exemplifies how bioarchaeological research, when bolstered by advanced laboratory diagnostics, can bring into focus the shifting epidemiological dynamics of infectious diseases in the Mediterranean basin. By analyzing dental pulp from individuals buried at the site of Mariana (9th−13th centuries CE), the authors identify direct evidence of Plasmodium spp., including *Plasmodium falciparum*, through paleo-auto-immunohistochemistry, metagenomics, and immunochromatographic assays. This work represents one of the most robust microbiological documentations of malaria in a European archaeological context. Its significance lies in both affirming and refining the historical record: confirming endemicity in Corsica long before the modern epidemiological data began and anchoring ancient perceptions of “bad air” (mal'aria) in a tangible biomedical framework. Importantly, the study advances the methodological scope of paleomicrobiology by validating dental pulp as a reliable substrate for detecting ancient intraerythrocytic pathogens.

Similarly engaged with malaria, though through a very different lens, is the discourse-historical study by Miao, which investigates the conceptual migration of malaria-related terms and knowledge into the Chinese medical lexicon during the late Qing and Republican periods. This article adds a rare linguistic-historiographical dimension to the issue. Miao traces how terms like *ague* and *malaria* were translated and embedded in Chinese as *nueji* and *zhangqi*, navigating epistemic tensions between Western parasitological models and traditional Chinese cosmological etiologies. The article identifies three key phases of terminological negotiation and medical reinterpretation, showing how newspapers and dictionaries became arenas for reconciling competing medical ontologies. Through this, Miao illustrates how the transmission of medical knowledge is not a one-way act of importation, but a transformative process of cultural re-codification. It is a timely reminder that the evolution of disease concepts cannot be fully understood without attention to linguistic and cultural mediation.

If malaria and its representations define the boundaries of infectious disease historiography in this Research Topic, the article by Tondini et al. on two ancient Egyptian skulls explores the limits of surgical and oncological intervention in antiquity. Held at the Duckworth Research Topic in Cambridge, the skulls—dating from the Old Kingdom and Late Period—reveal distinct pathological signatures: one evidencing healed cranial trauma, the other metastatic lytic lesions with perimortem cutmarks. These findings suggest not only the ability of ancient Egyptian physicians to manage severe head injuries but also the possible surgical exploration of malignant lesions. Employing micro-CT and microscopic analysis, the authors put forth the provocative idea that cancer, though often considered a diagnostic blind spot in ancient medicine, was perhaps recognized and acted upon in at least rudimentary form. The juxtaposition of these two cases—one evidencing therapeutic success, the other a potential attempt to confront malignancy—encapsulates the dual themes of limitation and innovation in early medical care. It also illustrates how paleopathology can meaningfully contribute to debates in the history of oncology.

From ancient Egypt to 18th-century Austria, Nerlich et al. revisit the enigmatic case of the so-called “air-dried chaplain” discovered in the crypt of St. Thomas am Blasenstein. What was once assumed to be a case of spontaneous mummification is revealed, through extensive interdisciplinary examination, to be a deliberate and elaborate embalming procedure. Through radiological, toxicological, and histological analyses, the authors reconstruct the post-mortem treatment of the body, including rectal packing with fabrics and wood, zinc- and arsenic-based chemical impregnation, and preservation likely driven by a mixture of religious reverence and early modern anatomical curiosity. Alongside these technical findings, the study reconstructs the chaplain's life history, suggesting high dietary status, a lack of heavy labor, and chronic pulmonary tuberculosis as a probable cause of death. The case offers a rare glimpse into regional mummification practices in early modern Europe, expanding paleopathology beyond the ancient world and reminding us that the impulse to preserve, investigate, and display the human body transcends historical epochs.

Finally, the review by Dadar et al. presents an ambitious and far-reaching history of brucellosis in the Middle East, spanning the Neolithic to modern times. Synthesizing paleopathological, textual, genetic, and epidemiological data, the authors trace the evolution of *Brucella* species, their zoonotic transitions, and their sociopolitical entrenchment in contemporary Middle Eastern public health. The strength of the article lies in its integration of “deep time” perspectives—from archaeological DNA and phylogenetic trees to modern One Health frameworks—demonstrating how long-term trajectories can inform present-day policies. Particularly illuminating is the discussion of how historical data can help target disease control strategies in a region where brucellosis remains endemic, with substantial socio-economic impacts. It is a compelling argument for embedding paleopathology into modern global health narratives.

Taken together, the five contributions to this volume span broad geographic, chronological, and methodological territories. Yet, they share a common epistemological thread: the conviction that diseases are not only biological phenomena but also deeply cultural, historical, and semiotic ones. These studies challenge the notion that pathology exists in a vacuum. They ask us to reconsider what it means to “diagnose” in absence of living patients, what it means to “treat” when medicine is guided by ritual or speculation, and what it means to “know” a disease across the centuries.

Paleopathology, in this sense, becomes more than the study of ancient ailments: it is a means of investigating the human condition itself—our fears, our interventions, our failures, and our astonishing capacity for resilience and adaptation. This Research Topic does not simply recover voices of the past; it situates them within conversations about the future of medical epistemology, translational science, and global health equity. Whether through a silent mummy, a fractured skull, or a misunderstood parasite, the past continues to speak—and this volume ensures that we are listening.
